# Insulin Sensitivity, Serum Lipids, and Systemic Inflammatory Markers in School-Aged Obese and Nonobese Children

**DOI:** 10.1155/2010/846098

**Published:** 2011-01-12

**Authors:** Jinkwan Kim, Rakesh Bhattacharjee, Leila Kheirandish-Gozal, Abdelnaby Khalyfa, Oscar Sans Capdevila, Riva Tauman, David Gozal

**Affiliations:** ^1^Department of Pediatrics, Comer Children's Hospital, The University of Chicago, Suite K-160, 5721, Maryland Avenue, MC 8000, Chicago, IL 60637, USA; ^2^Division of Sleep and Respiratory Medicine, Department of Pediatrics, The Hospital for Sick Children, University of Toronto, Toronto, ON, Canada M5G 1X8; ^3^Pediatric Sleep Unit, Division of Neurophysiology, Department of Neurology, Hospital Sant Joan de Déu, 08950 Barcelona, Spain; ^4^Dana Children's Hospital, Tel-Aviv University School of Medicine, Tel-Aviv 64239, Israel

## Abstract

The impact of obesity as a systemic low-grade inflammatory process has only partially been explored. To this effect, 704 community-based school-aged children (354 obese children and 350 age-, gender-, and ethnicity-matched controls) were recruited and underwent assessment of plasma levels of fasting insulin and glucose, lipids, and a variety of proinflammatory mediators that are associated with cardiometabolic dysfunction. Obese children were at higher risk for abnormal HOMA and cholesterol levels. Furthermore, BMI *z* score, HOMA, and LDL/HDL ratio strongly correlated with levels of certain inflammatory mediators. Taken together, obesity in children is not only associated with insulin resistance and hyperlipidemia, but is accompanied by increased, yet variable, expression of markers of systemic inflammation. Future community-based intervention and phenotype correlational studies on childhood obesity will require inclusion of expanded panels of inflammatory biomarkers to provide a comprehensive assessment of risk on specific obesity-related morbidities.

## 1. Introduction

Childhood obesity is a serious and progressively increasing public health problem that has reached epidemic proportions and in the United States disproportionately affects low-income and minority children [1–3]. Metabolic and cardiovascular complications of obesity in childhood, while less common than in adulthood, may nevertheless include insulin resistance and type 2 diabetes. Body mass index (BMI) tracks from childhood to adulthood and as such, overweight and obese children are at greater risk of developing not only hypertension, metabolic, and cardiovascular diseases, but also asthma and sleep apnea in later life [4–8].

In adults, obesity is associated with increases in systemic inflammatory markers, as evidenced by studies documenting the association of BMI and visceral obesity with circulating levels of cytokines and acute-phase reactants [9–11]. In children, the presence of obesity also appears to be associated with increased levels of high-sensitivity CRP (hsCRP) [12], as well as other inflammatory mediators [13–17], that promote the development of endothelial and metabolic dysfunction [18–21]. A recent review on this topic [22] concluded that although there appears to be sufficient evidence to support the existence of an association between obesity and increased hsCRP along with decreased adiponectin levels, the circulating levels found in the majority of the studies published in the literature are generally within the normative range, and could therefore underestimate the concentrations of these mediators at the tissue level. Tam et al. further recommended additional studies measuring IL-6 and TNF-*α*, as well as other interleukins and chemokines in young children [22]. 

Concurrent with such understanding of the published literature and in agreement with the recommendations by Tam and colleagues [22], we hypothesized that by examining the concentration of fasting morning plasma inflammatory mediators obtained from community-based obese children, we would expect to find variable expression of specific inflammatory mediators in this cohort that have previously been shown to increase the cardiovascular and/or metabolic disease risk.

## 2. Methods

### 2.1. Subjects

 The study was approved by the University of Louisville Human Research Committee, and informed consent was obtained from the legal caregiver of each participant. Consecutive children between the ages of 5 to 8 years attending public schools in Jefferson County, Louisville, KY, were invited to participate in the study, after they underwent a school-based health screening, which included height and weight measurements. Based on such screening, children were identified when their BMI *z* score was ≥1.65 and age- (within 3–6 months), gender-, ethnicity-, and area-of-residence-matched children with BMI *z* scores <1.65 were then identified and recruited to serve as controls. Of note, all children were otherwise healthy, were recruited from the community via the Jefferson County Public School Health Screening Program, and were representative of the demographic characteristics of the general population of the city of Louisville (http://ksdc.louisville.edu/sdc/census2000/cityprofiles/LouisvilleDP.pdf). Children were excluded if they had known diabetes or prediabetes (http://www.diabetes.org/pre-diabetes/pre-diabetes-symptoms.jsp), any defined genetic abnormality or underlying systemic disease including hypertension (as defined by a systolic or diastolic blood pressure exceeding the 95th percentile for age, gender, and height using population data obtained by the National Heart Lung and Blood Institute), or if they were within a month from any acute infectious process.

### 2.2. Anthropometry

 To verify the school-health screening initial reports, children were weighed using a calibrated scale to the nearest 0.1 kg and height (to 0.1 cm) was measured with a stadiometer (Holtain, Crymych, UK). Body mass index (BMI) was calculated and BMI z-score was computed using CDC 2000 growth standards (http://www.cdc.gov/growthcharts/) and online software (http://www.cdc.gov/epiinfo/). A BMI *z* score ≥1.65 was considered as fulfilling the criteria for obesity.

### 2.3. Blood Based Assays

Fasting blood samples were drawn by venipuncture in the morning. Blood samples were immediately centrifuged, and plasma was frozen at -80°C until assay. Plasma insulin levels were measured using a commercially available radioimmunoassay kit (Coat-A-Count Insulin; Diagnostic Products Inc). This method has a detection level of 1.2 *μ*IU/mL and exhibits linear behavior up to 350 *μ*IU/mL, with intra-assay and interassay coefficients of variability of 3.1% and 4.9%, respectively. Plasma glucose level was measured using a commercial kit based on the hexokinase-glucose-6-phosphate dehydrogenase method (Flex Reagent Cartridges; Dade Behring, Newark, DE). Insulin resistance was assessed using a widely validated mathematical formula, the homeostasis model assessment (HOMA) equation (fasting insulin × fasting glucose/22.5) [23].

Plasma hsCRP levels were measured within 2-3 hours after collection using the Flex Reagent Cartridge (Date Behring, Newark, DE). This method has a detection level of 0.05 mg/dL and exhibits linear behavior up to 255 mg/dL, with intra-assay and interassay coefficients of variability of 9% and 18%, respectively, for hsCRP. Serum lipids including total cholesterol, high-density lipoprotein (HDL) cholesterol, calculated low-density lipoprotein cholesterol (LDL), and triglycerides (TG) were also assessed using Flex Reagent Cartridges (Dade Behring). 

Plasma IL-6, IL-20, monocyte chemotactic protein (MCP), retinol-binding protein 4 (RBP4), and tumor necrosis factor (TNF-*α*) levels were measured using commercial ELISA kits (R&D systems, Minneapolis, MN). Plasma apolipoprotein B (ApoB), myeloid-related protein (MRP) 8/14, and macrophage inhibitory factor (MIF) levels were also measured using commercial ELISA kits (ALPCO Diagnostics, Salem, NH) following the manufacturer's instructions. Circulating levels of ICAM-1 and P-selectin were measured with commercially available kits (R&D System, Abington, UK). For ICAM-1, the sensitivity was 0.35 ng/mL and the intra- and interassay coefficients of variation were 2.5 and 1.8%, respectively. For P-selectin, the sensitivity was 0.5 ng/mL and the intra- and interassay coefficients of variation were 3.6 and 6.9%, respectively. All assays were performed in duplicate, and a calibration curve was included in each assay.

### 2.4. Statistical Analysis

 Data were expressed as mean ± SD. Significant differences within groups were analyzed using ANOVA followed by post-hoc tests with Bonferroni corrections for multiple comparisons for continuous variables and chi-square tests for categorical variables. Spearman's correlation analyses were conducted to examine potential associations between BMI and plasma concentrations of the various inflammatory mediators. Statistical analyses were performed using SPSS software (version 16.0; SPPS Inc., Chicago, Ill.). All *P* values reported are 2-tailed with statistical significance set at <.05.

## 3. Results

A total of 354 obese children and 350 age-, gender-, and ethnicity-matched nonobese children were recruited between May 2004 and October 2008. The demographic characteristics of this cohort are shown in Table 1 and are virtually identical to the published demographics of the city of Louisville, Kentucky. It should be pointed out that not every child could have all of the inflammatory markers assayed, as dictated by the limited amounts of plasma, such that, the number of inflammatory marker assays varied from one child to another. 

As anticipated, obese children had higher HOMA values, indicative of insulin resistance, and also exhibited higher LDL, VLDL, and TG levels and lower HDL concentrations compared to nonobese children (Table 1). 

Obese children also had significantly higher levels of hsCRP, IL-6, MRP 8/14, P-selectin, ICAM-1, IL-20, RBP4, MIF, and TNF-*α* compared to nonobese children (Table 2). Only ApoB and MCP showed similar levels among the 2 groups. Among the obese children, 57% showed one, 34% two, 19% three, and 15% four or more cardiometabolic biomarkers with elevated plasma levels.

Regression analyses between each of the inflammatory markers and actual BMI or BMI *z* score revealed significant correlations for the majority of these markers (Table 3). In addition, hsCRP was also significantly associated with IL-6 (*r* = 0.35; *n* = 145; *P* < .01), MRP 8/14 (*r* = 0.67; *n* = 236; *P* < .001), and with TNF-*α*  (*r* = 0.46; *n* = 122; *P* < .01). Furthermore, hsCRP was also positively correlated with ICAM-1 (*r* = 0.29; *n* = 98; *P* < .03) and with RBP4 (*r* = 0.32; *n* = 80; *P* < .05). Similarly, significant correlations emerged between RBP4, MRP 8/14, TNF-*α*, hsCRP, and HOMA (Table 3). In addition MRP 8/14, IL-6, hsCRP, MIF, P-selectin, ICAM-1, and TNF-*α* levels showed significant associations with LDL/HDL (Table 3).

## 4. Discussion

This study shows that systemic inflammatory processes are activated in otherwise asymptomatic, community-dwelling, obese, and school-age prepubertal children. Interestingly, the degree and nature of the activation of these inflammatory processes varied from child to child, and only a minority of obese children exhibited extensive derangements across multiple cardiometabolic inflammatory biomarkers. Furthermore, there were significant associations between specific subsets of the inflammatory markers and the degree of obesity, insulin resistance, or hyperlipidemia. However, while there was some degree of overlap among the inflammatory markers associated with each of these variables, the more remarkable finding was that even if a particular marker was elevated and correlated with end-organ dysfunction, this did not necessarily imply that all other markers were affected as well. The pathophysiology of end-organ morbidities that are traditionally perceived as resulting from the long-term effects of obesity on health has been postulated to involve low-grade activation of multiple pathways of inflammation. Based on the current understanding of the roles played by these inflammatory mediators, it becomes imperative to explore the role of genetic, environmental, and lifestyle influences on the modulation of the inflammatory responses in the context of obesity, and its consequences [24, 25]. Similarly, it will be critical to assess in the future the effect of interventions such as dietary changes and physical activity on the reversibility of these inflammatory responses and on the progression of obesity-related morbidities [26–30].

Before we discuss any further the potential implications of our findings, some technical and methodological approaches deserve comment. We selected a wide net array of known biomarkers for both cardiovascular and metabolic dysfunction, in an attempt to characterize the variability and the potential associations of these markers in the context of pediatric obesity. However, since hsCRP has been extensively used in past studies, we aimed to include this measure in all children. We also restricted the age range of our cohort to a narrow time span that is associated with the initial 3 years of attendance in the public school system, a period during which changes in eating patterns are now well described [31–34]. We also selected our population based on a representative community sample for the city of Louisville, Kentucky, and identified this cohort in the school system itself, rather than using a clinical referral cohort. As such, we also selected a closely matching nonobese child, in the same school, to control for and all of the potential confounders that could be introduced in the process of cohort allocation. However, neither pubertal status nor the presence of hepatic steatosis was assessed.

1Globally, the findings from this study indicate the presence of a substantial inflammatory burden in obese prepubertal children along with a high risk for insulin resistance and increased serum lipids. Therefore, our study is in close agreement with multiple other studies in children that have examined a selected number of inflammatory mediators in the context of obesity [12, 35–46]. Of note, in a study by Nagel and colleagues, these investigators did not find any evidence for a significant association between increased body weight in children and ApoB levels, and our current findings are in close concordance with such report in German children [44]. Similarly, our findings concur with the increased plasma levels of adhesion molecules in a pediatric overweight cohort from Mexico, suggesting evidence of endothelial dysfunction in a substantial proportion of obese children [47]. Of note, the inflammatory markers that were presently associated with either BMI, BMI *z* score, HOMA, and hypercholesterolemia varied, suggesting different and coordinated biological roles for clusters of inflammatory modulators. Indeed, although there was some degree of overlap among the inflammatory markers and their respective significant associations, only a portion of the markers were correlated with any given end-organ dysfunction. 

In summary, this study clearly shows that obesity in childhood carries a substantial inflammatory burden that is strongly, yet selectively, associated with specific functional alterations, such as insulin sensitivity or lipid homeostasis. With the emergence of multiplexed ELISA assays that exhibit improved sensitivities, we would advocate that future community-based intervention or correlational studies on childhood obesity [48] should explore more expansive panels of inflammatory markers along with functional phenotypes, and should also consider incorporation of genomic variance assessments using recently developed cardiovascular or metabolic gene-centric polymorphism arrays [49].

## Figures and Tables

**Table 1:** Demographic characteristics of obese and nonobese children.

	Obese (BMI *Z* ≥ 1.65)	Nonobese (BMI *Z* < 1.65)	*P*-value*
Age (years)	7.1 ± 0.4 (*n* = 354)	7.1 ± 0.4 (*n* = 350)	
Gender (% male)	51.2	51.1	
Ethnicity			
White Caucasian % (*n*)	77.9 (*n* = 277)	78.0 (*n* = 273)	
African-American % (*n*)	18.1 (*n* = 64)	18.0 (*n* = 63)	
BMI *z* score	2.21 ± 0.14	1.06 ± 0.21	<.00001
Systolic Blood Pressure (mmHg)	112.4 ± 9.7 (*n* = 354)	107.1 ± 10.9 (*n* = 350)	<.01
Diastolic blood pressure (mmHg)	67.1 ± 8.2 (*n* = 354)	62.7 ± 7.9 (*n* = 350)	<.01
Triglycerides	84.1 ± 5.1	73.5 ± 2.8	<.001
HDL	42.5 ± 1.8	62.9 ± 1.8	<.00001
VLDL	19.1 ± 1.3	14.7 ± 0.9	<.001
LDL	98.7 ± 2.9	93.3 ± 1.8	<.001
Glucose	81.3 ± 1.8	78.2 ± 1.7	<.04
Insulin	14.9 ± 1.7	6.7 ± 1.0	<.0001
HOMA-IR	2.3 ± 0.3	1.2 ± 0.3	<.0001

*Statistical significance determined using ANOVA test.

**Table 2:** Fasting morning plasma concentrations of inflammatory biomarkers in obese and nonobese children.

	Obese (BMI *Z* ≥ 1.65)	Nonobese (BMI *Z* < 1.65)	*P*-values*
MRP 8/14 (ug/mL)	1.5 ± 0.9 (*n* = 128)	0.9 ± 0.8 (*n* = 176)	.0001
Apo B (mg/dL)	77.8 ± 19.6 (*n* = 65)	73.7 ± 16.3 (*n* = 51)	.233
MCP (pg/mL)	132.0 ± 29.6 (*n* = 77)	120.1 ± 18.8 (*n* = 68)	.494
RBP4 (ng/mL)	33.4 ± 16.3 (*n* = 80)	18.2 ± 14.0 (*n* = 80)	.01
IL-20 (pg/mL)	4.0 ± 5.4 (*n* = 67)	2.3 ± 4.5 (*n* = 44)	.02
IL-6 (pg/mL)	1.8 ± 0.9 (*n* = 132)	1.0 ± 0.6 (*n* = 134)	.01
MIF (pg/mL)	188.6 ± 103.1 (*n* = 132)	131.7 ± 86.4 (*n* = 106)	.02
hsCRP (mg/dL)	2.3 ± 1.4 (*n* = 354)	0.5 ± 0.3 (*n* = 350)	<.0001
TNF-*α* (pg/mL)	598.5 ± 62.2 (*n* = 172)	317.3 ± 42.2 (*n* = 176)	.0001
P selectin (ng/mL)	94.3 ± 22.2 (*n* = 56)	37.7 ± 12.9 (*n* = 90)	.0001
ICAM-1 (ng/mL)	397.4 ± 83.5 (*n* = 56)	247.4 ± 53.5 (*n* = 90)	.0001

*Statistical significance determined using ANOVA test.

**Table 3:** Correlation coefficients between BMI and BMI *z* score and plasma levels of several inflammatory markers in children.

	BMI	BMI *z* score	HOMA	LDL/HDL ratio
	*r*-value	*r*-value	*r*-value	*r*-value
MRP 8/14 (ug/mL) (*n* = 249)	0.354*	0.375*	0.332*	0.227*
Apo B (mg/dL) (*n* = 116)	0.143	0.160	0.02	0.10
MCP (pg/mL) (*n* = 143)	0.00	0.00	0.00	0.12
RBP4 (ng/mL) (*n* = 160)	0.213*	0.317*	0.376*	0.09
IL-20 (pg/mL) (*n* = 111)	−0.054	−0.036	0.01	0.03
IL-6 (pg/mL) (*n* = 266)	0.161*	0.177*	0.132*	0.134*
MIF (pg/mL) (*n* = 108)	0.043	0.02	0.110*	0.176*
hsCRP (mg/dL) (*n* = 704)	0.472*	0.367*	0.276*	0.365*
TNF-*α* (pg/mL) (*n* = 348)	0.298*	0.336*	0.187*	0.215*
P selectin (ng/mL) (*n* = 146)	0.310*	0.376*	0.087	0.387*
ICAM-1 (ng/mL) (*n* = 146)	0.254*	0.276*	0.104	0.413*

In parenthesis in the number of observations for which data were available,

*P < .05. Statistical significance determined using Spearman's correlational analysis.
